# Effect of chronic kidney disease on all-cause mortality in tuberculosis disease: an Australian cohort study

**DOI:** 10.1186/s12879-022-07039-5

**Published:** 2022-02-02

**Authors:** Beau Z. Carr, Esther M. Briganti, Joseph Musemburi, Grant A. Jenkin, Justin T. Denholm

**Affiliations:** 1 Department of Infectious Diseases, Monash Health, Melbourne, Australia; 2 Department of Epidemiology and Preventive Health, Monash University, Melbourne, Australia; 3 Victorian Tuberculosis Program, Melbourne Health, Melbourne, Australia; 4 Department of Infectious Diseases, University of Melbourne, Parkville, Australia

**Keywords:** Chronic kidney disease, CKD, Diabetes, Mortality, TB, Tuberculosis

## Abstract

**Background:**

While there has been a recent epidemiological and clinical focus on the interaction between diabetes and tuberculosis, the interaction between chronic kidney disease and tuberculosis has been less studied. In particular, little is known of the effect of eGFR levels well above that seen in end stage kidney disease on mortality.

**Methods:**

We conducted a retrospective cohort study of 653 adults from a large Australian hospital network, using data from a state-wide registry of reported tuberculosis cases between 2010 and 2018, with ascertainment of diabetes status and renal function data from hospital medical records and laboratory data. Cox proportional hazards regression models were used to calculate hazard ratios for all-cause mortality associated with categories of chronic kidney disease in adults with tuberculosis disease.

**Results:**

Total number of deaths was 25 (3.8%). Compared to tuberculosis cases with eGFR ≥ 60 ml/min, all-cause mortality was higher for those with chronic kidney disease from an eGFR level of 45 ml/min. The association was independent of sex, age and diabetes status with adjusted hazard ratio of 4.6 (95% CI: 1.5, 14.4) for eGFR 30–44 ml/min and 8.3 (95% CI: 2.9, 23.7) for eGFR < 30 ml/min.

**Conclusions:**

Our results suggest a notably increased risk of all-cause mortality even in those with more moderate degrees of renal impairment, in a low tuberculosis prevalence setting. The impact of these findings on a population basis are at least as significant as that found with diabetes and warrant further investigation in populations with higher tuberculosis prevalence.

**Supplementary Information:**

The online version contains supplementary material available at 10.1186/s12879-022-07039-5.

## Background

There is an increasing burden of non-communicable diseases, including diabetes and chronic kidney disease (CKD), in countries with both high and low burdens of tuberculosis (TB). The interaction of diabetes and TB in relation to the presentation and outcomes of both conditions has been a focus of a number of epidemiological and clinical studies in the last decade. This has resulted in the development of health care guidelines by several international public health institutions relating to the care and control of diabetes and TB.

People requiring dialysis are known to be at a significantly increased risk of infection, including TB [1]. This has been attributed to associated immune dysfunction, as well as shared negative socio-economic factors and clinical co-morbidities, including diabetes [2]. The risk of TB in the dialysis population has been shown to reflect the prevalence of TB in the country of birth of individuals, suggesting reactivation of latent TB infection acquired before dialysis initiation, rather than infection after the onset of dialysis [3]. The relative impact of dialysis on TB infection and disease is also complicated by the association of diabetes with end stage renal disease, however there is relatively little published data to allow disaggregation of the relative impact of these conditions.

There is emerging evidence of susceptibility to infection in lesser degrees of renal impairment, in terms of infection-related hospitalisation and subsequent mortality [4–6]. This has been particularly observed for septicaemia, and pulmonary and genitourinary infections [7]. Studies attempting to elucidate the underlying pathophysiology of this increased risk of infection suggest that the immune dysfunction relates mainly to cell-mediated rather than humoral immunity [8]. Impaired cell-mediated immunity is known to have an important role in TB reactivation from latent infection. Despite the potential global relevance of CKD and TB comorbidity, especially for many low and medium-income countries where there is a convergence of CKD and TB epidemics, this association has been largely overlooked [9]. Only two large cohort studies, both from Taiwan, a country with intermediate TB risk, have examined the relationship between CKD and TB disease [10, 11]. Both identified an increased incidence of TB disease in CKD, and in the study where eGFR data was collected, the risk was evident at an eGFR of 45 ml/min [11]. Few studies have addressed the effect of lesser degrees of renal impairment on the outcomes of TB disease, specifically all-cause mortality. We therefore aim to examine the association of CKD with all-cause mortality in a low TB prevalence setting.

## Methods

### Data source

All adults (aged 18 years or above) with TB disease managed between 1 January 2010 and 31 December 2018 within a large Australian hospital network were eligible for inclusion. Data was collected from a comprehensive state-wide registry of TB cases at the Victorian Tuberculosis Program, to which reporting from clinicians and laboratories is mandatory. Case definition of TB disease emphasises culture or polymerase chain reaction confirmation of *Mycobacterium tuberculosis* (typically > 85% of cases in this setting), and also includes clinical or radiological diagnosis by a medical practitioner experienced in TB management.

### Co-morbidities

Demographic (sex, age and country of birth), clinical (previous history of TB, site of the disease [pulmonary, extra-pulmonary and pulmonary plus other site/s]) and microbiological (basis of diagnosis [culture based or not] and drug resistance [pan-susceptible, resistant to at least one drug but not multidrug resistant or multidrug resistant]) data was obtained from the registry. Information relating to renal status (based on eGFR at diagnosis) and diabetes status (based on clinician reported diabetes in the medical records or laboratory HbA1c greater than 6.5% at TB diagnosis) was collected by interrogation of medical records and laboratory data at Monash Health. Kidney function was categorised as eGFR ≥ 60 ml/min, 45–59 ml/min, 30–44 ml/min and < 30 ml/min.

### Outcomes

Death within 12 months of notification was available from the registry. In terms of specific causes of death, for all cases who died before or during treatment, registry case notes were independently reviewed and adjudicated in regards to the degree to which TB was likely to have contributed to death according to established definitions [12]. If TB was the primary cause of death or contributed to death, or the cause of death was unknown, the cause of death was classified as being TB related. Otherwise the cause of death was classified as being due to other causes, but specific causes of death were not available.

The primary outcome of interest was all-cause mortality across the four kidney function categories. The secondary outcome of interest was cause-specific (TB related and non-TB related) mortality, however due to the lack of TB related deaths in two of the four eGFR categories (45–59 ml/min and 30–44 ml/min), the analysis was performed using two kidney function categories of ≥ 30 ml/min and < 30 ml/min.

### Statistical analyses

Categorical variables were expressed as frequencies and percentages, and continuous variables as means and standard deviations. Pearson’s chi square test was used to assess for associations between categorical variables, and independent Student’s t-test was used to test for associations between continuous data.

In terms of the primary outcome, a Cox proportional hazard model was used to compare all-cause mortality across the four categories of kidney function. The model was adjusted by sex and age (model 1), and sex, age and diabetes status (model 2). Interactions between categories of kidney function and sex, age and diabetes status for all-cause mortality were examined. In terms of the secondary outcome of cause-specific mortality, the competing risks regression methods of Fine and Gray was used. Survival time was calculated from the date of notification to the date of death from any cause. For TB cases who were not known to have died or had died after 12 months of follow-up, time of follow-up was censored on the last date they were known to be alive, with follow-up limited to 12 months. The assumption of proportional hazard was confirmed with the use of Schoenfeld residuals. Results are presented as hazard ratio (HR) with 95% confidence interval (95% CI).

The level of significance for all analyses was 0.05. Statistical analyses were conducted using Stata version 14 (Stata Corp, College Station, TX, USA).

### Ethics statement

Data collected by the Victorian Tuberculosis Program and used under the legislative authority of the Public Health and Wellbeing Act 2008, did not require Human Research Ethics Committee approval as per the rules of the institution. Approval by data managers from the Victorian Department of Health and Human Services was obtained for this review. For data collected from medical records at Monash Health, this study was considered a Quality & Service Improvement activity and therefore also did not require Human Research Ethics Committee approval (Monash Health Reference: RES-18-0000-745Q).

## Results

### Baseline characteristics

**Fig. 1:**
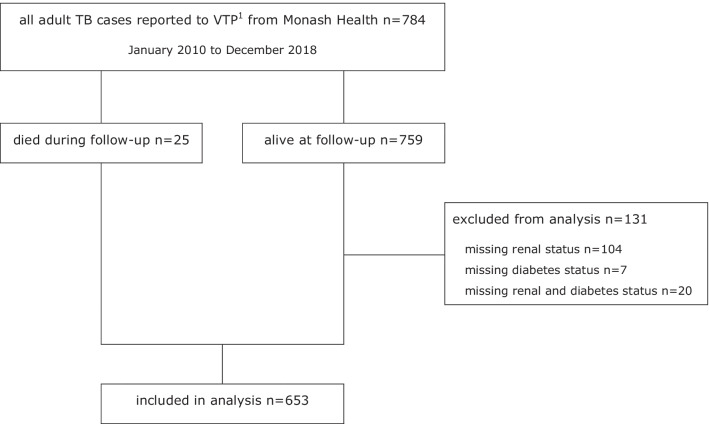
Flow diagram for study population inclusion and exclusion

For the study period 2010–2018, 784 cases were identified as having been diagnosed or treated at Monash Health, representing just over 30% of all adult cases in the state of Victoria (784/2550). Data related to diabetes status (n = 7), renal function (n = 104) or both (n = 20) was not able to be ascertained from the medical records or laboratory data in 131 (16.7%) cases as shown in Fig. 1. Type of diabetes was not available from the medical records in the majority of cases. The number of cases on dialysis was 8 (1.0%). The primary analysis included 653 cases.

Antibiotic sensitivities were available for 515/520 (99.0%) of cases diagnosed based on positive culture results.

Differences in demographic and clinical characteristics between cases included and those not included due to missing data are shown in Table 1. Those excluded from the study due to missing exposure data (eGFR or diabetes status) were more likely to be younger (< 65 years of age), Australian-born, not have a culture-based diagnosis or not have diabetes.

**Table 1:** Demographic and clinical characteristics of included and non-included TB cases

	Included	Excluded	P-value
Total	653 (83.3)	131 (16.7)	
Sex, male	360 (55.1)	70 (53.4)	0.722
Age, years	44.3 ± 19.8	37.7 ± 17.4	< 0.000
Age, ≥ 65 years	125 (19.1)	11 (9.4)	0.012
Country of birth, overseas	635 (97.2)	114 (87.0)	0.000
High TB prevalence of country of birth	461 (70.6)	84 (64.1)	0.142
Previous TB	37 (5.7)	7 (5.3)	0.884
Site of disease			
Pulmonary	307 (47.0)	68 (51.9)	0.098
Pulmonary plus other site/s	102 (15.6)	11 (8.4)	
Extra-pulmonary	244 (37.4)	52 (39.7)	
Culture-based diagnosis	520 (79.6)	90 (68.7)	0.006
Drug resistance^a^			
Pan-susceptible	463 (89.0)	80 (88.9)	0.139
Resistant to at least one drug, but not MDR-TB	49 (9.4)	6 (6.7)	
MDR-TB	8 (1.5)	4 (4.4)	
eGFR^b^, mls	83.6 ± 16.1	88.6 ± 3.8	0.413
eGFR categories^b^			
≥ 60 ml/min	602 (92.1)	7 (100.0)	0.898
45–59 ml/min	18 (2.6)	0 (0.0)	
30–44 ml/min	15 (2.4)	0 (0.0)	
< 30 ml/min	18 (2.9)	0 (0.0)	
Diabetes status^c^, yes	82 (12.6)	5 (4.8)	0.021

Mean ± SD; n (%)

^a^For culture positive cases only (n = 520 included cases, n = 90 excluded cases)

^b^eGFR data available in only 7 of excluded cases

^c^Diabetes status data available in only 104 of excluded cases

### Mortality and associated demographic, clinical and microbiological characteristics

Total number of deaths was 25 (3.8%). Demographic and clinical characteristics were available for all cases, and microbiological characteristics only for those with a culture-based diagnosis are shown in Table 2. Of the deaths, 8/25 were recorded as TB related and the remaining 17/25 as non-TB related.

**Table 2:** Demographic and clinical characteristics of TB cases

		All	Survived	Died^a^
Total		653	628	25 (4.0)
Sex	Female	293	286	7 (2.4)
	Male	360	342	18 (5.0)
Age	< 65 years	528	523	5 (1.0)
	≥ 65 years	125	105	20 (16.0)
Country of birth	Australia	18	18	0 (0.0)
	Overseas	635	610	25 (4.1)
TB prevalence of COB^b^	Low	192	182	10 (5.2)
	High	461	446	15 (3.3)
Previous TB	No	616	592	24 (3.9)
	Yes	37	36	1 (2.7)
Site of disease	Pulmonary	307	292	15 (4.9)
	Pulmonary plus other site/s	102	96	6 (5.9)
	Extra-pulmonary	244	240	4 (1.6)
Culture-based diagnosis	No	133	126	2 (1.5)
	Yes	520	497	23 (4.4)
Drug resistance^c^	Pan-susceptible	463	441	22 (4.8)
	Resistant to at least one drug, but not MDR-TB	49	48	1 (2.0)
	MDR-TB	8	8	0 (0.0)
Renal impairment, eGFR	≥ 60 ml/min	602	592	10 (1.7)
	45–59 ml/min	18	16	2 (11.1)
	30–44 ml/min	15	10	5 (33.3)
	< 30 ml/min	18	10	8 (44.4)
Diabetes status	No	571	556	15 (2.6)
	Yes	82	72	10 (12.2)

^a^n (%)

^b^Country of birth

^c^For culture positive cases only (n = 520)

### Risk of all-cause mortality

Strong associations were found between age, diabetes and renal function for all-cause mortality as shown in Table 3. The association between diabetes and all-cause mortality was largely accounted for by older age, with the hazard ratio dropping from 4.8 (95% CI: 2.2–10.8, P < 0.000) to 1.5 (95% CI: 0.7–3.5, P = 0.301) after adjusting for age, with no further impact after also adjusting for sex, with hazard ratio of 1.5 (95% CI: 0.7–3.4, P = 0.349). All-cause mortality notably increased with lower levels of eGFR, the hazard ratio being as high as 22.1 (95% CI: 7.6, 64.8 P < 0.000) for eGFR between 30–44 ml/min, and 34.0 (95% CI: 13.4, 86.3, P < 0.00) for eGFR < 30 ml/min. While these hazard ratios were attenuated after adjusting for age and sex, they remained significant. Further adjustment for diabetes did not change the magnitude of the hazard ratios. No interaction was found between CKD and age, sex or diabetes status for all-cause mortality at any of the eGFR cut off points (all P > 0.05).

**Table 3:** All-cause mortality in TB cases

	Univariable		Adjusted^a^		Adjusted^b^	
	Hazard ratio (95% CI)	P value	Hazard ratio (95% CI)	P value	Hazard ratio (95% CI)	P value
Sex: males versus females	2.1 (0.9, 5.1)	0.090	2.1 (0.9, 5.0)	0.108	2.1 (0.9, 5.0)	0.107
Age, per 10 years	2.2 (1.7, 2.8)	< 0.000	1.9 (1.4, 2.5)	0.000	1.9 (1.4, 2.5)	0.000
Diabetes status: yes versus no	4.8 (2.2, 10.8)	< 0.000	–	–	0.9 (0.4, 2.2)	0.855
Renal function:						
≥ 60 ml/min	REF.		REF.		REF.	
45–59 ml/min	7.1 (1.6, 32.5)	0.011	1.1 (0.2, 5.5)	0.884	1.1 (0.2, 5.5)	0.867
30–44 ml/min	22.1 (7.6, 64.8)	< 0.000	4.6 (1.5, 14.4)	0.009	4.6 (1.5, 14.4)	0.003
< 30 ml/min	34.0 (13.4, 86.3)	< 0.000	8.2 (3.0, 22.5)	< 0.000	8.3 (2.9, 23.7)	< 0.000

^a^Adjusted by age (per 10 years) and sex: model 1

^b^Adjusted by age (per 10 years), sex and diabetes status: model 2

In terms of other demographic, clinical and microbiological characteristics, only site of disease was found to be associated with all-cause mortality. The presence of synchronous involvement of pulmonary and extra-pulmonary sites was associated with higher all-cause mortality compared to the presence of extra-pulmonary disease alone (HR 3.6 (95% CI: 1.0–12.9, P = 0.046). However, the magnitude of this association was reduced and became non-significant once accounting for age (adjusted HR 1.7 (95% CI: 0.5–6.1, P = 0.438). Adjusting for the site of disease, with or without the addition of age, sex and diabetes status in the model, did not change the magnitude or statistical significance of the association between kidney function and all-cause mortality. Of the deaths, 23 were amongst the 520 cases with a culture-based diagnosis-22/23 (95.7%) had fully sensitive TB, 1/23 (4.3%) had isoniazid resistant TB, and none had multi-resistant TB.

The population attributable fraction of CKD on all-cause mortality in TB cases was 57% for eGFR < 60 ml/min, 49% for eGFR < 45 ml/min and 31% for eGFR < 30 ml/min, compared to 31% for diabetes.

### Risk of cause specific mortality

Strong associations were found between age, diabetes and eGFR < 30 ml/min with both the risk of TB related and non-TB related cause specific mortality as shown in Additional file 1: Table S1 and Additional file 2: Table S2, respectively. The effect of eGFR < 30 ml/min was attenuated after adjusting for sex, age and diabetes status, but remained large and significant for both causes of mortality.

## Discussion

This study found that within a cohort of adults with TB disease in a low TB prevalence setting, with 12.6% having diabetes and just under 7.9% being identified with CKD defined as an eGFR less than 60 ml/min, renal impairment was very strongly associated with all-cause mortality. Furthermore, there was a notable increasing effect of lower levels of eGFR on all-cause mortality, even after accounting for important confounding factors of age and diabetes, at least from an eGFR level of 45 ml/min. The negative effect of CKD on all-cause mortality was similar for those with and without diabetes.

Previous studies have identified CKD as a risk factor for the development of TB disease in countries with intermediate [10, 11] and high [13, 14] prevalence of TB. The relative magnitude of the risk from CKD in the cohort studies was small, with relative risks of less than 2, similar to the magnitude of risk seen with diabetes in the same studies [10, 11]. This study has demonstrated a strong association between CKD and all-cause mortality, the hazard ratio ranging from 4.6 to 8.3 with increasing severity of CKD, greater than that seen for the association between CKD and the development of TB disease in the previously published studies. Furthermore, severe renal impairment is specifically associated with TB related mortality.

Recognition of the negative impact of CKD not only on the development of end stage kidney disease but also on cardiovascular disease outcomes emphasises the importance of global efforts to prevent its development and progression [15]. The increasing prevalence of CKD in middle- and low-income countries is particularly relevant due to the shared high TB prevalence in many of these countries [16]. Furthermore, there is also the emergence of diabetes as the main cause of CKD globally. While the focus of the interaction between TB and diabetes remains relevant, our results suggest that the contribution of CKD defined as eGFR less than 60 ml/min on all-cause mortality in TB disease is nearly double that of diabetes, the population attributable fraction being 57% and 31%, respectively. The population attributable fraction of a more clinically significant degree of renal impairment of an eGFR less than 30 ml/min on all-cause mortality, is the same as that of diabetes among those with TB disease in this study at 31%. These findings highlight the importance of effective screening, prevention and early treatment of CKD in tackling not only the development of TB disease but also the mortality associated with it.

In Australia, the burden of CKD (eGFR less than 60 ml/min) in the general population is at least similar to that of diabetes, which have been reported at about 5% [17, 18]. More than 80% of the general Australian population with renal impairment do not, however, have diabetes [19]. This underscores the novel findings of this study in terms of the importance of considering renal impairment as an important risk factor for mortality in those with TB disease, independent of diabetes, as well as amongst those with eGFR levels much higher than in those with end stage kidney disease. The implications of these findings are likely to be particularly impactful in countries of more highly endemic TB, where the co-epidemic of diabetes and CKD is emerging, and health care resources may be limited.

Guidelines currently recommend systematic testing and treatment of latent tuberculosis in certain high-risk groups, which includes people on dialysis, to reduce the burden of active tuberculosis and consequent risk of mortality, even in countries with low TB burden. Our study supports the importance of such screening and subsequent treatment, and provides strong support for further studies of the potential benefit of expanding this recommendation to those with less severe renal impairment, particularly in countries with high prevalence of CKD.

The quantitatively larger effect of severe renal impairment on TB related mortality compared to non-TB related mortality may reflect the compounding effects of factors, such as malnutrition and immunosuppression, that are seen in both severe renal impairment and TB disease. It is also possible that in the setting of renal impairment, selection or dose-adjustment of anti-tuberculosis medications may potentially be resulting in under-treatment of TB disease and therefore greater susceptibility to TB related mortality. Our findings support the need for randomised controlled trials to provide evidence to guide TB treatment in those with renal impairment, as well as pharmacokinetic studies to better evaluate the possibility of under-treatment and inform dose optimisation.

Despite this study’s important and novel clinical findings, there are a number of limitations.

1. The study was an analysis of data collected retrospectively from a single metropolitan healthcare service. However, in terms of generalisability of the findings to the whole of Australia, despite this cohort represented only 30% of total Victorian TB cases, patient characteristics and outcomes were similar to those previously reported for all Victorian cases [20]. In addition, Victorian TB cases are largely representative of Australia more broadly [21].
2. Incidence rate of TB disease in Australia is one of the lowest in the world at 5.3 cases per 100,000 population in 2015. Generalisability of our findings to other countries with higher TB burden may therefore be limited. However, the majority of cases in this study were born overseas, mainly from countries of high TB prevalence. Assuming that the strength of the association between CKD and all-cause mortality seen in this study is similar for middle- and low-income countries, where not only TB disease but also CKD is prevalent, these findings are particularly relevant for the potential of CKD becoming a driving force to increasing mortality related to TB in these countries.
3. Chronic kidney disease was defined by a single baseline eGFR value at the time of diagnosis of TB disease and was based on eGFR alone as data on proteinuria was not available in the majority of cases. Possible misclassification in terms of renal function, such that those with CKD may have been over-estimated due to negative effects of illness on renal function, would only strengthen the relationship between lower eGFR and all-cause mortality. In addition, many anti-tuberculosis medications can be nephrotoxic, but we were unable to address the impact of renal impairment during the treatment period on all-cause mortality, due to incomplete availability of renal function results after TB diagnosis.
4. The presence of diabetes as an associated diagnosis was reliant on accurate recording in the medical record, and therefore was likely to have been under-represented. In addition, the degree of glycaemic control and impact of other diabetes related complication on clinical outcome was not able to be fully ascertained. However, it is likely that those with the most significant presentation of diabetes were more likely to have the diagnosis recorded in the medical notes and had tests ordered to assess glycaemic control by HbA1c. Therefore, the observed lack of independent effect of diabetes on all-cause mortality in this study is unlikely to have been an under-estimate of the risk from diabetes. The limited documentation of glycaemic control as assessed by HbA1c did not allow for the assessment of degree of hyperglycaemia on all-cause mortality.
5. Information relating to clinical factors known to impact on all-cause mortality such as socioeconomic, HIV and nutritional status, associated substance abuse and severity of TB infection (22) was either unknown or only available for a limited number of the cohort, so could not be examined.

## Conclusions

In conclusion, this study found that CKD, with eGFR levels well above that seen in end stage kidney disease, was associated with a significantly greater risk of all-cause mortality, as well as specific TB related mortality, in people with TB disease. These associations were seen at least from an eGFR of 45 ml/min for all cause mortality and eGFR of 30 ml/min for TB related mortality, and were independent of sex, age and the presence of diabetes. These findings need to be confirmed in prospective studies of larger populations of people with TB disease, with more accurate and detailed characterisation of diabetes and CKD to allow for more confident verification of the association observed in this retrospective study. Global public health implications of these findings also necessitate the urgent undertaking of corroborating studies examining the association between CKD and mortality in moderate to high prevalence countries for TB. Mechanisms underlying this association, as well as that of an increased risk of TB infection reported in other studies, require clearer elucidation. Focus should be on the pathogenic mechanisms implicated in the increased risk of mortality, the accuracy of current screening and diagnostic testing for TB disease, as well as efficacy and safety of antibiotic treatment in the setting of renal impairment. Our findings, suggest that effective strategies to help reduce the burden of both CKD and diabetes, both already common and increasingly prevalent non-communicable diseases world-wide, may assist in global efforts to reduce mortality in those with TB disease. Multi-disciplinary and integrated health care strategies that deliver optimal healthcare to those with TB disease and CKD, may improve outcomes and reduce mortality.

## Supplementary Information

**Additional file 1: Table S1**. Competing Risk Analysis For TB Related Mortality.

**Additional file 2: Table S2**. Competing Risk Analysis For Non-TB related Mortality.

## Data Availability

The datasets analysed during the current study are available from the corresponding author on reasonable request.

## Abbreviations

CI: Confidence intervals
CKD: Chronic kidney disease
HR: Hazard ratio
SHR: Subdistribution hazard ratio
TB: Tuberculosis
