# Supplementary material for: Effect of chronic kidney disease on all-cause mortality in tuberculosis disease: an Australian cohort study
Source: BMC Infect Dis. 2022 Feb 2;22:116. doi: 10.1186/s12879-022-07039-5 (PMC8812263; doi:10.1186/s12879-022-07039-5)
Supplement: Supplementary file 1 — Additional file 1: Table S1. Competing Risk Analysis For TB Related Mortality. [file 12879_2022_7039_MOESM1_ESM.docx]

Additional File 1. Competing Risk Analysis for TB Related Mortality

|  | Univariable |  |  | Adjusted^1^ |  |  | Adjusted^2^ |  |
| --- | --- | --- | --- | --- | --- | --- | --- | --- |
|  | SHR (95% CI) | P value |  | SHR (95% CI) | P value |  | SHR (95% CI) | P value |
| Sex: males versus females | 1.36 (0.32, 5.69) | 0.673 |  | 0.99 (0.21, 4.65) | 0.993 |  | 0.99 (0.21, 4.65) | 0.993 |
| Age, per 10 years | 2.93 (2.02, 4.24) | 0.000 |  | 2.60 (1.61, 4.19) | 0.001 |  | 2.60 (1.63, 4.12) | 0.000 |
| Diabetes status: yes versus no | 7.11 (1.79, 28.31) | 0.005 |  |  |  |  | 0.99 (0.18, 5.47) | 0.993 |
| Renal function: ≥30 ml/min  <30 ml/min | REF  39.30 (10.29, 150.03) | 0.000 |  | REF  9.59 (2.42, 38.09) | 0.001 |  | REF  9.62 (1.80, 51.49) | 0.008 |

SHR subdistribution hazard ratio

1. Model 1: adjusted by age (per 10 years) and sex

2. Model 2: adjusted by age (per 10 years), sex and diabetes status
