# Supplementary material for: Effect of chronic kidney disease on all-cause mortality in tuberculosis disease: an Australian cohort study
Source: BMC Infect Dis. 2022 Feb 2;22:116. doi: 10.1186/s12879-022-07039-5 (PMC8812263; doi:10.1186/s12879-022-07039-5)
Supplement: Supplementary file 2 — Additional file 2: Table S2. Competing Risk Analysis For Non-TB related Mortality. [file 12879_2022_7039_MOESM2_ESM.docx]

Additional File 2. Competing Risk Analysis for Non-TB Related Mortality

|  | Univariable |  |  | Adjusted^1^ |  |  | Adjusted^2^ |  |
| --- | --- | --- | --- | --- | --- | --- | --- | --- |
|  | SHR (95% CI) | P value |  | SHR (95% CI) | P value |  | SHR (95% CI) | P value |
| Sex: males versus females | 2.69 (0.88, 8.23) | 0.083 |  | 2.30 (0.75, 7.05) | 0.146 |  | 2.30 (0.74, 7.10) | 0.148 |
| Age, per 10 years | 1.96 (1.63, 2.34) | 0.000 |  | 1.84 (1.51, 2.23) | 0.000 |  | 1.84 (1.49, 2.27) | 0.000 |
| Diabetes status: yes versus no | 3.85 (2.2, 10.8) | 0.008 |  |  |  |  | 0.98 (0.37, 2.60) | 0.970 |
| Renal function: ≥30 ml/min  <30 ml/min | REF  11.76 (3.92, 35.26) | 0.000 |  | REF  3.32 (1.01, 10.92) | 0.048 |  | REF 3.34 (1.10, 10.16) | 0.033 |

SHR subdistribution hazard ratio

1. Model 1: adjusted by age (per 10 years) and sex

2. Model 2: adjusted by age (per 10 years), sex and diabetes status
